# Pharmacokinetics and efficacy of a novel long-acting bupivacaine formulation for cornual nerve block in calves

**DOI:** 10.3389/fvets.2022.1060951

**Published:** 2022-12-01

**Authors:** Dinakaran Venkatachalam, Nikki Kells, Paul Chambers, Antony Jacob, Neil Ward, Preet Singh

**Affiliations:** School of Veterinary Science, Massey University, Palmerston North, New Zealand

**Keywords:** pharmacokinetics, efficacy, long-acting bupivacaine formulation, cornual nerve block, calves

## Abstract

Local anesthetics are commonly used in farm animals to provide analgesia for painful procedures but can cause adverse effects at high systemic concentrations. The pharmacokinetics and efficacy of a long-acting sucrose acetate isobutyrate (SAIB) bupivacaine formulation following cornual nerve block in calves were compared to lidocaine. Fourteen calves were randomly assigned to one of the treatment groups (i) 5% Bupivacaine-SAIB (BUP-SAIB), *n* = 7; or (ii) 2% lidocaine (LID), *n* = 7. Cornual nerve block was performed, and duration of effective analgesia was evaluated by nociceptive threshold testing using a hand-held pressure algometer. Blood samples were collected at various time points and plasma concentrations were analyzed by HPLC. Pharmacokinetic parameters were calculated using a non-compartmental model. The mechanical nociceptive thresholds showed that the novel formulation could desensitize the skin around the horn bud for 18.77 ± 8.88 h (range 8–36 h), compared to 0.79 ± 0.34 h (range 0.5–1.5 h) with lidocaine. The mean maximum plasma concentration (C_max_) of bupivacaine was 152.03 (SD 37.34) ng/mL and its T_max_ was 0.39 (SD 0.13) h. The half-life of elimination was 32.79 ± 11.00 h and the rate of clearance was 0.12 ± 0.03 L h^−1^. No toxicity signs were seen after treatment in either group. The novel formulation produced long-lasting analgesia of several times greater duration than that produced by lidocaine. This study showed that the safety and efficacy of the SAIB formulation justifies further studies in a larger population of animals.

## Introduction

Farm animals are routinely subjected to painful husbandry procedures that may significantly affect their welfare if performed without pain relief. Local anesthetic nerve block techniques are the most common and cost-effective methods used for alleviating husbandry pain in farm animals (1). These techniques are simple to perform, cheaper and relatively safer than general anesthetics. Although nerve blocks can effectively eliminate intraoperative pain during painful procedures, they have minimal effect on post-operative pain because of their short duration of action (2). Pain following most painful husbandry procedures can last for several hours to weeks (3–5).

Disbudding is a routine procedure performed in most dairy herds around the world (6). Whilst cornual nerve block using local anesthetics such as lidocaine can eliminate pain during disbudding, its short duration of action makes it unsuitable for managing post-disbudding pain (7). Inadequate postoperative pain management can affect the well-being of the animal and can also delay wound healing (8). Multimodal analgesia using lidocaine and meloxicam is commonly used to manage pre- and post-operative disbudding pain (9, 10). However, meloxicam reduces but does not abolish post-disbudding pain (11). In addition, meloxicam does not mitigate the acute pain that is experienced once the local anesthetic effects wear off (12). Developing a local anesthetic formulation that could abolish both perioperative and postoperative pain from a single injection would be a simple and useful option for farm animals.

Bupivacaine is a potent long-acting local anesthetic that can provide local anesthesia for several hours (13). Prolonging the duration of action of bupivacaine could be a good option to manage perioperative and postoperative pain. There are a number of ways to maintain sufficient local anesthetic concentrations at the nerve for an extended time (14, 15) such as simply injecting higher concentrations of local anesthetics in the same volume. Increasing the concentration of the local anesthetics has been shown to increase the duration of action but this will also increase the risk of systemic toxicity (14) as a higher total dose is given. Alternatively, the duration of local anesthesia can be prolonged without causing systemic toxicity by formulating local anesthetics in a sustained release formulation. Sustained release formulations reduce the risk of systemic toxicity by reducing the rate of absorption of the drugs and thus systemic concentrations (15).

Sucrose acetate isobutyrate (SAIB) is a highly viscous and hydrophobic biodegradable sugar. It has been used as a matrix for the sustained release of drugs such as deslorelin acetate and bupivacaine (16, 17). Mixing a small quantity of solvent (ethanol or N-methyl pyrrolidone) with SAIB reduces its viscosity by several fold, making it suitable for injection (18). Drug formulations containing SAIB form an *in-situ* gel following injection into tissues and release the drugs slowly. We hypothesized that using a SAIB-based bupivacaine formulation would release bupivacaine gradually and prolong its duration of local anesthesia without causing toxicity.

The objectives of this study were to develop a novel sustained release formulation of bupivacaine and compare its local anesthetic efficacy with the commonly used local anesthetic lidocaine hydrochloride following cornual nerve block in calves; and to investigate the pharmacokinetics of bupivacaine in calves to ensure that toxic plasma concentrations were not reached.

## Materials and methods

### Drugs and chemicals

Bupivacaine free base and articaine hydrochloride were purchased from Wuhan Hezhong Biochemical Manufacturing Co. Ltd, (Wuhan, China) and SCI Pharmtech (Taoyuan, Taiwan), respectively. Lidocaine hydrochloride (Lopaine 2%) was purchased from Ethical agents Ltd., Auckland, New Zealand. Sucrose acetate isobutyrate (90%) in ethanol, *N*-Methylpyrrolidone (NMP) and acetonitrile were purchased from Merck, Auckland, New Zealand. Heparin sodium and normal saline were purchased from Pfizer New Zealand Limited (Auckland, New Zealand) and Baxter Healthcare Pty Ltd., (Old Toongabbie, NSW, Australia), respectively. Reagent grade potassium dihydrogen phosphate was obtained from Merck KGaA (Darmstadt, Germany). Phosphoric acid was obtained from Thermo Fisher Scientific Ltd (Scoresby, VIC, Australia). Milli-Q water was produced by the Milli-q PFplus system (Millipore Corporation, Bedford, MA, USA).

### Preparation of bupivacaine-SAIB formulation

Appropriate quantities of SAIB and NMP were weighed in a beaker and mixed in a vortex. To this mixture, bupivacaine was added and vortexed again for 10 min. Various formulations containing different ratios of SAIB: NMP and 5% bupivacaine were prepared and the release of bupivacaine from each formulation was investigated *in vitro* using the dialysis method (unpublished data). The formulation that showed the most prolonged drug release properties *in vitro* was studied in calves (unpublished data). The final composition of the formulation used in this study was 73% SAIB, 22% NMP and 5% bupivacaine (W/W/W).

### Experimental animals

A group of fourteen Friesian X Hereford calves (3–4 weeks old, male) were purchased from a private farm (Rongotea, New Zealand) and transported in a covered trailer to the experimental facility. A sample size of seven was selected based on data comparing the duration of sensory blockade in sheep following brachial plexus nerve block with different local anesthetics (19). The authors reported mean durations of sensory blockade of 100 ± 38 and 335 ± 134 min for lidocaine and bupivacaine, respectively. Based on a power of 90% and type 1 error rate of 5%, the required sample size was calculated as 4. To allow for any exclusions due to illness e.g., calf diarrhea, and to account for the expected high individual variations in plasma drug concentration in test calves (20), a total of 14 calves (*n* = 7 per treatment) were enrolled, with the aim of collecting at least 6 data sets per group. Animals were sourced from a commercial farm as close as possible to the research facility in order to minimize transport duration, in accordance with the refinement principle of the three R's. Only calves that were healthy (based on physical examination) and had horn buds were used. Upon arrival, calves were identified with numeric ear tags and housed as a single group in a pen bedded with wood shavings. They were fed two liters of milk replacer (Milligans Classic, Milligans Feed Ltd., Oamaru, New Zealand) twice daily and had *ad libitum* access to water and calf pellets (Earlywean 16% Calf Pellets, Sharpes Farm Feeds, Carterton, New Zealand). Massey University Animal Ethics Committee approved the experimental protocol (MUAEC 20–12).

### Experimental design and procedure

This study consisted of two groups: (i) Bupivacaine-SAIB formulation block (BUP-SAIB) and (ii) commercial 2% lidocaine hydrochloride block (LID) to represent current practice. Seven calves were assigned to each group. After a week of acclimatization, each calf was subjected to a clinical examination by a veterinarian. Calves were then weighed (mean 46 ± 5 kg, range 40–51.5 kg) and randomly allocated to either the BUP-SAIB (*n* = 7) or LID (*n* = 7). Calves were tested in two cohorts on the 5th (calves 1, 4, 7, 8, 10, 12, 14) and 10th (calves 2, 3, 5, 6, 9, 11, 13) of November 2020. Treatment allocation and day of testing were determined using a random number generator. On the day of the treatment, 2 h after their morning feed, test calves were moved into a separate pen and catheters were placed in the right jugular vein of the BUP-SAIB group to facilitate repeat blood sampling. The catheterization procedure was as follows: calves were gently manually restrained and the area over the jugular vein was clipped and surgically prepared with povidone-iodine. The catheter site was subcutaneously infiltrated with 1 mL of 4% articaine hydrochloride prior to making a stab incision in the skin over the vein with a #22 scalpel blade. A 14G × 130 mm catheter (BD Insyte, Becton Dickinson Medical, Sandy, UT, USA) was then inserted into the right jugular vein and sutured to the skin using 3/0 monofilament nylon suture. The patency of the catheter was maintained by flushing with 2 mL heparin saline solution (10 U/ml units heparin sodium in saline). The time between catheterization and nerve block ranged from 45 min to 1 h.

Calves were gently restrained and the hair around the horn buds was clipped. Cornual nerve block was performed on both sides by the same experienced veterinarian and the efficacy of nerve block was evaluated using a hand-held digital algometer. The BUP-SAIB group received Bupivacaine-SAIB formulation (5% bupivacaine) and the LID group received 2% lidocaine hydrochloride solution. The cornual nerves were blocked by injecting 5 mL of the local anesthetic subcutaneously (20 G; 25 mm needle) below the caudal ridge of the supraorbital process halfway between the horn bud and lateral canthus of the eye. The right cornual nerve was blocked followed by the left cornual nerve.

Mechanical nociceptive testing was carried out at four sites around each horn bud (rostal, caudal, lateral, and medial) at 0 (prior to injection), 5, 15, and 30 min and 1, 1.5, 2, 4, 6, 8, 10, 12, 24, 36, and 48 h after drug administration using a handheld pressure algometer (FPX 25, Wagner Instruments, Greenwich, CT, USA) with a 4 mm-diameter round stainless-steel tip. One researcher (with whom the calves were familiar) gently manually restrained the calf and held the head whilst a second researcher carried out the testing. The probe was placed gently on the horn bud without applying force. If calves reacted immediately to the placement of the probe the procedure was repeated. If calves did not respond immediately to the placement of the probe force was applied and manually increased until either the animal responded (any attempted movement of the head away from the probe or head shaking was considered a positive withdrawal response) or a predetermined cut-off force was reached (12.0 N). The minimum value recorded prior to treatment (baseline) was 3.2 N and the maximum was 12.0 N. The same evaluator, who was blinded to treatments, carried out the mechanical nociceptive testing for all calves. Calves remained in the pen with their cohort for the duration of testing.

Blood samples (5 mL in heparinized tubes) were collected from BUP-SAIB calves at 0 (prior to administration), 15, 30 min and 1, 2, 4, 6, 8, 12, 24, 36, and 48 h after administration of the formulation for analysis of plasma bupivacaine concentration. Approximately 2 mL of blood was withdrawn from the catheter in a separate syringe (to be discarded) before collection of the sample for analysis. The catheter was then flushed with 2 mL of heparinized saline. In the event of loss of catheter or blockage, blood samples were collected directly from the opposite jugular vein. Immediately after collection, blood samples were cooled on ice and plasma was separated and stored at −20°C.

Animals were observed for signs of pain or local anesthetic toxicity such as sedation, ataxia, and convulsion during injection and at 0.5, 1, 2, 4, 6, 8, 24, 36, and 48 h following injection. At the end of the study, calves were euthanized by captive bolt stunning followed by exsanguination and the site of injection was observed for adverse effects.

### Determination of plasma concentrations

The concentrations of bupivacaine in the plasma samples collected following the administration of the bupivacaine-SAIB formulation were measured using a modified HPLC method previously reported by Danielsson et al. (21). Standard stock solutions (1 mg/mL) of bupivacaine were prepared by dissolving the bupivacaine in methanol, and the working standard solutions were prepared by serially diluting the standard stock solutions in water. Calibration standards (50–1,000 ng/ml) and quality control samples (50, 250, and 1,000 ng/mL) were freshly prepared by adding working standard solutions to pooled calf plasma collected from calves before treatment.

The HPLC analysis was performed in a Shimadzu chromatographic system (Shimadzu Corporation, Japan) equipped with a binary pump, column thermostat and diode array detector. Chromatographic separation was achieved using a C_18_ reversed-phase column (Synergi Hydro-RP Column 250 × 4.6 mm, 4 μm; Phenomenex Inc., Torrance, CA, USA) maintained at 40°C. The mobile phase consisted of a mixture of phosphate buffer (10 mM, 1.36 g potassium dihydrogen phosphate in 1 L water; pH 4.0 adjusted using orthophosphoric acid) and acetonitrile (70:30, v/v) and was delivered at a flow rate of 1 mL/min.

The plasma samples were processed by the protein precipitation method. A 200 μL aliquot of plasma was taken in a 2 mL microcentrifuge tube and 800 μL of ice-cold methanol was added and vortexed for 2 min. After 10 min, the samples were vortexed again and centrifuged at 4,500 *g* (Thermo Fisher Scientific, Jiangsu, China) for 10 min. The supernatant (800 μL) was then transferred to phospholipid removal tubes (Phree tubes; Phenomenex Inc., Torrance, CA, USA) placed in a glass tube and centrifuged at 250 *g* for 4 min. The collected eluent in the glass tubes was then evaporated in a vacuum rotary evaporator (70°C). The dried samples were then reconstituted in the mobile phase (200 μL) and injected into HPLC. The volume of the injectate was 100 μL, the column temperature was 40 °C and the ultraviolet wavelength was set at 210 nm.

### Pharmacokinetic analysis

Pharmacokinetic parameters of bupivacaine were calculated using non-compartmental analysis. Individual animal pharmacokinetic parameters were calculated using PKSolver add-on (22) for Excel 2010 (Microsoft, Redmond, CA, USA). The rate constant of the terminal phase (λ_z_) was estimated by linear regression of the logarithmic plasma concentration and the terminal half-life (t_1/2λ*z*_) as determined using the formula t_1/2λ*z*_ = 0.693/λ_z_. The maximum concentration in plasma (C_max_) and time to reach C_max_ (T_max_) were determined using PKSolver. The area under the curve (AUC) and the area under the first moment curve (AUMC) was determined using the linear trapezoidal method. Mean residence time was calculated as:

$$\begin{array}{l} MRT=AUMC/AUC \end{array}$$

The results are reported as mean ± S.D.

## Results

The nerve block was effective within 5 min in all except one calf (BUP-SAIB group) that reacted to the algometer testing throughout the testing period. Data from this calf were excluded from subsequent analysis. The total duration of nerve block as determined by MNT testing for individual animals is presented in Table 1. The mean duration of nerve block observed in the BUP-SAIB and LID group was 18.17 and 0.79 h, respectively. The maximum duration of nerve block produced by the novel formulation and lidocaine hydrochloride was 36 and 1.25 h, respectively. Raw data from individual animals is available in Supplementary material.

**Table 1:** Duration of cornual nerve block (hours) following administration of bupivacaine-SAIB formulation (5% bupivacaine, *n* = 7) and lidocaine hydrochloride (2% lidocaine hydrochloride, *n* = 7).

**Animals**	**BUP-SAIB**		**LID**	
	**Right horn**	**Left horn**	**Right horn**	**Left horn**
1	10.0	24.0	1.0	0.5
2	10.0	24.0	1.0	0.5
3	8.0	10.0	0.5	1.0
4	36.0	12.0	1.5	1.25
5	12.0	24.0	0.5	0.5
6	*NE*	*NE*	0.5	1.0
7	24.0	24.0	1.0	0.5
Mean	18.17		0.79	
SD	8.88		0.34	

NE, no effect (excluded from analysis).

**Figure 1:**
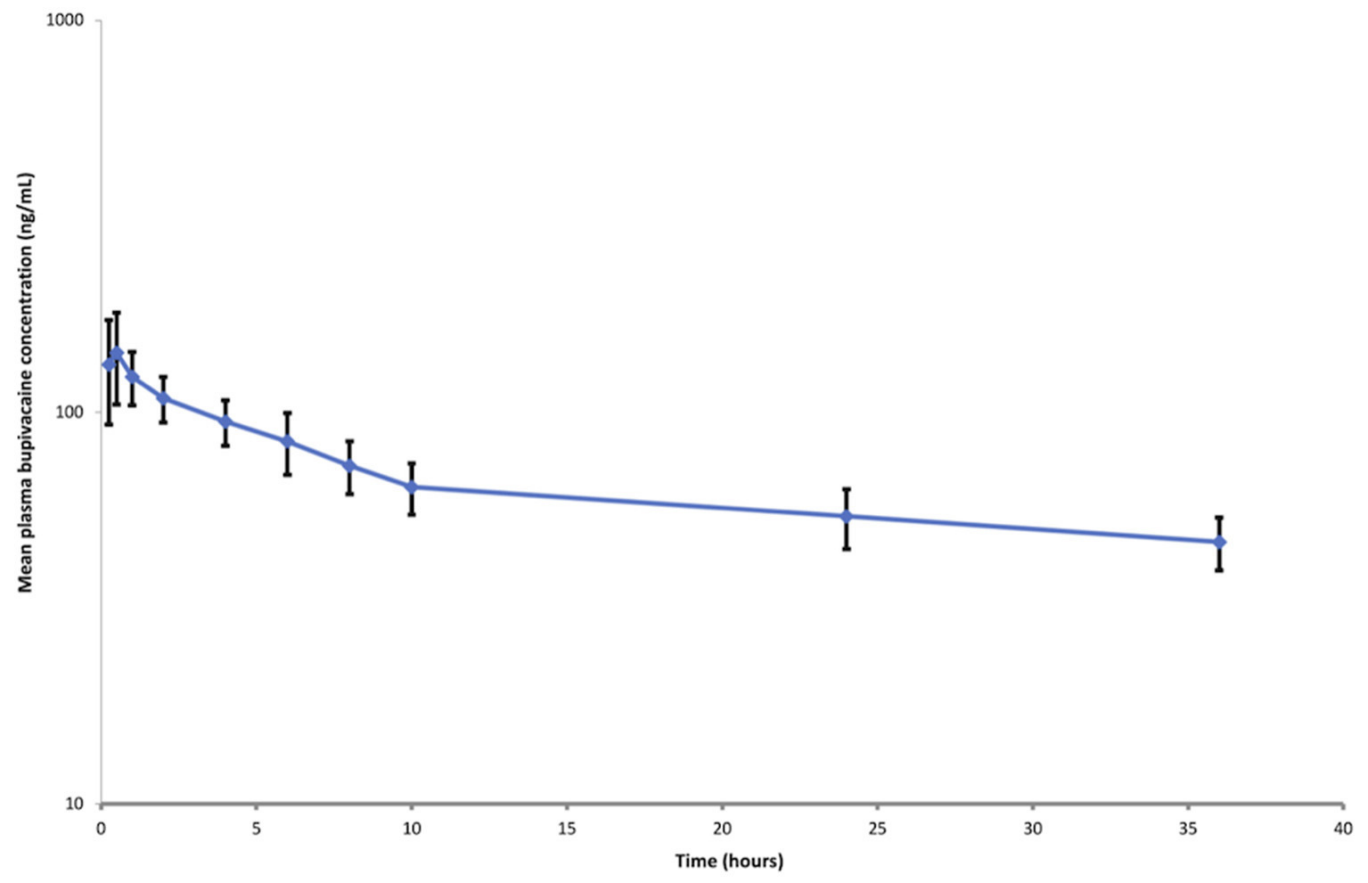
Semi-logarithmic plot of plasma bupivacaine concentration (mean ± standard deviation) vs. time following cornual nerve block using BUP-SAIB formulation (5 mL per site; total dose, 500 mg) in seven calves.

The calibration curves (50–1,000 ng/ml) of bupivacaine were linear with correlation coefficient values (*r*^2^) above 0.9957. The lower limit of quantification (LLOQ) of bupivacaine was 50 ng mL^−1^. The intra- and inter-day coefficients of variation (% CV) were ≤10.35 and ≤14.34%, respectively. The extraction recoveries of bupivacaine were more than 71.85%. Semi-logarithmic graph depicting the plasma bupivacaine concentration vs. time following cornual nerve block using BUP-SAIB formulation is shown in Figure 1. The pharmacokinetic parameters of bupivacaine following cornual nerve block are listed in Table 2. The mean maximum concentration (C_max_) of bupivacaine was 152.03 (SD 37.34) ng/mL, achieved at 0.39 (SD 0.13) h_._ The half-life of elimination was 32.79 ± 11.00 h and the rate of clearance was 0.12 ± 0.03 L h^−1^.

**Table 2:** Pharmacokinetic parameters (mean ± SD) of bupivacaine following cornual nerve block using BUP-SAIB formulation (5 mL per side) in calves (*n* = 7).

**Parameters**	**BUP-SAIB (*n* = 7)**
C_max_ (ng mL^−1^)	152.03 ± 37.34
T_max_ (h)	0.39 ± 0.13
t_½λz_ (h)	32.79 ± 11.00
AUC (h ng mL^−1^)	4,257.35 ± 976.97
MRT (h)	46.50 ± 14.40
CL (L h^−1^)	0.12 ± 0.03

C_max_, maximum plasma concentration; T_max_, time of C_max_; AUC, area under the curve; MRT, mean residence time; t_½λz_ elimination half-life; CL, clearance rate.

Slight swelling around the injection site was observed in most of the calves that received the novel formulation. The swelling remained during the observation period (48 h) and was not painful to touch. Gross pathological examination revealed slight erythematous lesions at the injection sites and the presence of the injected formulation. No such side effects were seen in the lidocaine group.

## Discussion

This study compared the local anesthetic efficacy of a novel sustained release formulation of bupivacaine with the commonly used local anesthetic lidocaine hydrochloride following cornual nerve block in calves. Our findings demonstrated that the novel bupivacaine formulation could provide long-lasting analgesia (18.77 ± 8.88 h) compared to the commonly used 2% lidocaine hydrochloride injection (0.79 ± 0.34 h). The MNT results suggest that the novel formulation can desensitize the skin around the horn buds for a maximum of 36 h with a range of 8 to 36 h. The variation in the duration of local anesthesia observed between and within animals could be due to the variation in the amount of formulation deposited around the nerves. It is likely that the anatomical location of the nerve and associated connective tissue varied between and within calves. Significant variations in the local anesthetic duration have been previously reported in calves following cornual nerve block using 2% lidocaine hydrochloride (7).

Bupivacaine 0.5% is considered to have a delayed onset of action (20 min) compared to lidocaine 2%. The novel bupivacaine formulation used in this study produced desensitization within 5 min. An initial burst release of bupivacaine, the higher concentration (5%) and the presence of the free base form of bupivacaine (unionized form) in the formulation could have led to the rapid onset of anesthesia.

A novel liposomal formulation of bupivacaine (Nocita^®^) has been reported to effectively control disbudding pain similar to the combined administration of lidocaine nerve block and oral meloxicam (23). The long-lasting anesthesia observed in the present study suggests that the novel formulation produces effective anesthesia similar to that of the liposomal bupivacaine formulation. However, it must be noted that Martin et al. (23) measured MNT following disbudding whereas no disbudding was carried out in this study. Disbudding is likely to cause sensitization. The authors also used other parameters such as infrared thermography, pressure mat gait analysis and behavior scoring, and serum cortisol and prostaglandin E2 metabolites to determine the anesthetic efficacy. Therefore, future studies should test the anesthetic efficacy of the novel formulation following disbudding and should include other parameters such as behavioral, physiological, and hormonal parameters.

Cornual nerve block was effective in all but one calf that received the novel formulation. This could be because the formulation was not deposited near the cornual nerves, or the horn buds of the calf were supplied with a cutaneous branch of cervical nerve two (C2) (24). No investigation was carried out to determine the cause. Cornual nerve block was not repeated in that calf as the administration of additional doses would have affected the plasma concentrations.

The plasma concentrations of bupivacaine were seen to be above the lower limit of the quantification (50 ng/mL) for up to 36 h following the administration of the formulation. The concentration vs. time curve showed no rapid decline in the plasma concentrations. These prolonged bupivacaine plasma concentration levels observed over time suggest that bupivacaine was gradually released from the BUP-SAIB formulation from the site of injection. The long elimination half-life and slow clearance of bupivacaine observed in this study were also reported following the administration of other long-acting bupivacaine formulations (7). In dogs and humans, the plasma concentrations of bupivacaine that are associated with central nervous system toxicity are in the range of 2–4 μg/mL (25), although there are no data in calves. The C_max_ (152.03 ± 37.34 ng/mL) observed in this study is well below the reported toxic plasma concentration indicating that the risk of systemic toxicity is low with this formulation. However, further studies on a larger number of animals are required to confirm the safety of this formulation.

In countries such as those in the European Union, where lidocaine is no longer approved for use in food-producing animals, the much older local anesthetic procaine is used instead. Procaine has been shown to be less potent than lidocaine, with slower onset and duration of anesthesia (26) and is unlikely to provide much post-operative analgesia. Although bupivacaine is the most widely used local anesthetic in people in most countries worldwide, it is not commonly approved for animals. Procaine is not the drug of choice for long-acting analgesia, but other ester type local anesthetics may be suitable for formulation with SAIB.

The excipients used in the novel formulation were non-pharmaceutical grade so a contaminant could have caused the swelling and erythematous lesions at the injection sites. It must be noted that the swelling did not appear to be painful to touch, and no pain-related signs were observed in any of the calves. It is likely that this was an osmotic effect. Further research is required to investigate the side effects of the novel formulation. SAIB is a safe biodegradable sugar used as an excipient to prolong the release of various drugs in people. A SAIB-based bupivacaine formulation (Posimir^®^) was recently approved for infiltration into the subacromial space to provide post-operative analgesia. Posimir^®^ has been reported to provide post-surgical analgesia for up to 72 h in humans. The concentration of bupivacaine in Posimir^®^ is 13.1% which is greater than the concentration of bupivacaine used in the novel formulation (5%). It is possible that increasing the concentration of bupivacaine in the novel formulation can further increase the duration of anesthesia.

The results of this study suggest that the novel BUP-SAIB formulation can prolong the duration of local anesthesia by gradually releasing bupivacaine from the injection site. Based on mechanical nociceptive threshold testing, the novel formulation provided several fold greater duration of anesthesia compared to the commonly used lidocaine nerve block. Sustained-release local anesthetic formulations for peripheral nerve blocks and infiltration are gaining popularity in human and veterinary medicine because of their ability to effectively control both pre and post-operative pain without major side effects. In addition to disbudding, this formulation could also be investigated for other procedures requiring peripheral nerve blocks or local infiltration such as velvet antler removal, tail docking and castration. This study provided preliminary information on the efficacy and safety of the novel formulation for cornual nerve block in calves. More work is needed to determine the physicochemical properties, safety, residue levels and efficacy of this formulation in calves undergoing disbudding.

## Conclusions

Based on nociceptive threshold data, the novel BUP-SAIB formulation provided a long-lasting cornual nerve block compared to the commonly used lidocaine hydrochloride with minimal side effects. The formulation released bupivacaine for a prolonged duration from the injection site, but plasma concentrations were always well below toxic levels. Further investigation in a larger population is indicated.

## Data availability statement

The original contributions presented in the study are included in the article/Supplementary material, further inquiries can be directed to the corresponding author.

## Ethics statement

The animal study was reviewed and approved by the Massey University Animal Ethics Committee (approval number 20/12).

## Author contributions

DV, NK, PC, NW, and PS were responsible for the conception of the study, data collection, data analysis, and manuscript preparation. AJ and DV were responsible for HPLC validation and sample analysis. All authors contributed to the article and approved the submitted version.

## Funding

This study was supported by Massey University Research Funds (MURF).

## Conflict of interest

The authors declare that the research was conducted in the absence of any commercial or financial relationships that could be construed as a potential conflict of interest.

## Publisher's note

All claims expressed in this article are solely those of the authors and do not necessarily represent those of their affiliated organizations, or those of the publisher, the editors and the reviewers. Any product that may be evaluated in this article, or claim that may be made by its manufacturer, is not guaranteed or endorsed by the publisher.
