# Supplementary material for: Pharmacokinetics and efficacy of a novel long-acting bupivacaine formulation for cornual nerve block in calves
Source: Front Vet Sci. 2022 Dec 1;9:1060951. doi: 10.3389/fvets.2022.1060951 (PMC9751437; doi:10.3389/fvets.2022.1060951)
Supplement: Supplementary file 1 [file Table_1.DOCX]

**Pharmacokinetics and efficacy of a novel long-acting bupivacaine formulation following cornual nerve block in calves**

**Supplementary data: Results of mechanical nociceptive threshold testing of individual calves**

KEY

Baseline = prior to cornual nerve block

NR = no response before predetermined cut off point of 12 N

✓ = withdrawal response to force between 3.2 and 12 N

(3.2 N was the minimum force at which a withdrawal response was observed prior to nerve block in any calf)

BUP-SAIB = novel bupivacaine formulation

LID = commercial 2% lidocaine hydrochloride

| **Calf ID** | 1 | **Treat** | BUP-SAIB | |  |  |  |  |
| --- | --- | --- | --- | --- | --- | --- | --- | --- |
|  | **Right horn bud** | | | | **Left horn bud** | | | |
| **Time** | **cranial** | **caudal** | **medial** | **lateral** | **cranial** | **caudal** | **medial** | **lateral** |
| Baseline | 3.6 | 4.3 | 5.8 | 6.7 | 5.3 | 7.1 | 4.5 | 6.2 |
| 5 mins | NR | NR | NR | NR | NR | NR | NR | NR |
| 15 mins | NR | NR | NR | NR | NR | NR | NR | NR |
| 30 mins | NR | NR | NR | NR | NR | NR | NR | NR |
| 1 hour | NR | NR | NR | NR | NR | NR | NR | NR |
| 1.5 hour | NR | NR | NR | NR | NR | NR | NR | NR |
| 2 hours | NR | NR | NR | NR | NR | NR | NR | NR |
| 4 hours | NR | NR | NR | NR | NR | NR | NR | NR |
| 6 hours | NR | NR | NR | NR | NR | NR | NR | NR |
| 8 hours | NR | NR | NR | NR | NR | NR | NR | NR |
| 10 hours | NR | NR | NR | NR | NR | NR | NR | NR |
| 12 hours | ✓ | ✓ | ✓ | ✓ | NR | NR | NR | NR |
| 24 hours | ✓ | ✓ | ✓ | ✓ | NR | NR | NR | NR |
| 36 hours | ✓ | ✓ | ✓ | ✓ | ✓ | ✓ | ✓ | ✓ |
| 48 hours | ✓ | ✓ | ✓ | ✓ | ✓ | ✓ | ✓ | ✓ |

| **Calf ID** | 2 | **Treat** | LID |  |  |  |  |  |
| --- | --- | --- | --- | --- | --- | --- | --- | --- |
|  | **Right horn bud** | | | | **Left horn bud** | | | |
| **Time** | **cranial** | **caudal** | **medial** | **lateral** | **cranial** | **caudal** | **medial** | **lateral** |
| Baseline | 3.7 | 6.3 | 8.3 | 6.8 | 10.2 | 6.9 | 12 | 6.5 |
| 5 mins | NR | NR | NR | NR | NR | NR | NR | NR |
| 15 mins | NR | NR | NR | NR | NR | NR | NR | NR |
| 30 mins | NR | NR | NR | NR | NR | NR | NR | NR |
| 1 hour | NR | NR | NR | NR | ✓ | ✓ | ✓ | ✓ |
| 1.5 hour | ✓ | ✓ | ✓ | ✓ | ✓ | ✓ | ✓ | ✓ |
| 2 hours | ✓ | ✓ | ✓ | ✓ | ✓ | ✓ | ✓ | ✓ |
| 4 hours | ✓ | ✓ | ✓ | ✓ | ✓ | ✓ | ✓ | ✓ |
| 6 hours | ✓ | ✓ | ✓ | ✓ | ✓ | ✓ | ✓ | ✓ |
| 8 hours | ✓ | ✓ | ✓ | ✓ | ✓ | ✓ | ✓ | ✓ |
| 10 hours | ✓ | ✓ | ✓ | ✓ | ✓ | ✓ | ✓ | ✓ |
| 12 hours | ✓ | ✓ | ✓ | ✓ | ✓ | ✓ | ✓ | ✓ |
| 24 hours | ✓ | ✓ | ✓ | ✓ | ✓ | ✓ | ✓ | ✓ |
| 36 hours | ✓ | ✓ | ✓ | ✓ | ✓ | ✓ | ✓ | ✓ |
| 48 hours | ✓ | ✓ | ✓ | ✓ | ✓ | ✓ | ✓ | ✓ |

| **Calf ID** | 3 | **Treat** | LID |  |  |  |  |  |
| --- | --- | --- | --- | --- | --- | --- | --- | --- |
|  | **Right horn bud** | | | | **Left horn bud** | | | |
| **Time** | **cranial** | **caudal** | **medial** | **lateral** | **cranial** | **caudal** | **medial** | **lateral** |
| Baseline | 4.6 | 5.5 | 7.2 | 8.2 | 7.2 | 5.7 | 11.1 | 4.8 |
| 5 mins | NR | NR | NR | NR | NR | NR | NR | NR |
| 15 mins | NR | NR | NR | NR | NR | NR | NR | NR |
| 30 mins | NR | NR | NR | NR | NR | NR | NR | NR |
| 1 hour | NR | NR | NR | NR | ✓ | ✓ | ✓ | ✓ |
| 1.5 hour | ✓ | ✓ | ✓ | ✓ | ✓ | ✓ | ✓ | ✓ |
| 2 hours | ✓ | ✓ | ✓ | ✓ | ✓ | ✓ | ✓ | ✓ |
| 4 hours | ✓ | ✓ | ✓ | ✓ | ✓ | ✓ | ✓ | ✓ |
| 6 hours | ✓ | ✓ | ✓ | ✓ | ✓ | ✓ | ✓ | ✓ |
| 8 hours | ✓ | ✓ | ✓ | ✓ | ✓ | ✓ | ✓ | ✓ |
| 10 hours | ✓ | ✓ | ✓ | ✓ | ✓ | ✓ | ✓ | ✓ |
| 12 hours | ✓ | ✓ | ✓ | ✓ | ✓ | ✓ | ✓ | ✓ |
| 24 hours | ✓ | ✓ | ✓ | ✓ | ✓ | ✓ | ✓ | ✓ |
| 36 hours | ✓ | ✓ | ✓ | ✓ | ✓ | ✓ | ✓ | ✓ |
| 48 hours | ✓ | ✓ | ✓ | ✓ | ✓ | ✓ | ✓ | ✓ |

| **Calf ID** | 4 | **Treat** | BUP-SAIB | |  |  |  |  |
| --- | --- | --- | --- | --- | --- | --- | --- | --- |
|  | **Right horn bud** | | | | **Left horn bud** | | | |
| **Time** | **cranial** | **caudal** | **medial** | **lateral** | **cranial** | **caudal** | **medial** | **lateral** |
| Baseline | 4.6 | 3.2 | 4.6 | 3.8 | 4.1 | 5.6 | 4.4 | 3.8 |
| 5 mins | NR | NR | NR | NR | NR | NR | NR | NR |
| 15 mins | NR | NR | NR | NR | NR | NR | NR | NR |
| 30 mins | NR | NR | NR | NR | NR | NR | NR | NR |
| 1 hour | NR | NR | NR | NR | NR | NR | NR | NR |
| 1.5 hour | NR | NR | NR | NR | NR | NR | NR | NR |
| 2 hours | NR | NR | NR | NR | NR | NR | NR | NR |
| 4 hours | NR | NR | NR | NR | NR | NR | NR | NR |
| 6 hours | NR | NR | NR | NR | NR | NR | NR | NR |
| 8 hours | NR | NR | NR | NR | NR | NR | NR | NR |
| 10 hours | NR | NR | NR | NR | NR | NR | NR | NR |
| 12 hours | ✓ | ✓ | ✓ | ✓ | NR | NR | NR | NR |
| 24 hours | ✓ | ✓ | ✓ | ✓ | NR | NR | NR | NR |
| 36 hours | ✓ | ✓ | ✓ | ✓ | ✓ | ✓ | ✓ | ✓ |
| 48 hours | ✓ | ✓ | ✓ | ✓ | ✓ | ✓ | ✓ | ✓ |

| **Calf ID** | 5 | **Treat** | LID | |  |  |  |  |
| --- | --- | --- | --- | --- | --- | --- | --- | --- |
|  | **Right horn bud** | | | | **Left horn bud** | | | |
| **Time** | **cranial** | **caudal** | **medial** | **lateral** | **cranial** | **caudal** | **medial** | **lateral** |
| Baseline | 4.6 | 5.3 | 6.6 | 4.9 | 7.8 | 6.3 | 5.7 | 4.8 |
| 5 mins | NR | NR | NR | NR | NR | NR | NR | NR |
| 15 mins | NR | NR | NR | NR | NR | NR | NR | NR |
| 30 mins | NR | NR | NR | NR | NR | NR | NR | NR |
| 1 hour | ✓ | ✓ | ✓ | ✓ | NR | NR | NR | NR |
| 1.5 hour | ✓ | ✓ | ✓ | ✓ | ✓ | ✓ | ✓ | ✓ |
| 2 hours | ✓ | ✓ | ✓ | ✓ | ✓ | ✓ | ✓ | ✓ |
| 4 hours | ✓ | ✓ | ✓ | ✓ | ✓ | ✓ | ✓ | ✓ |
| 6 hours | ✓ | ✓ | ✓ | ✓ | ✓ | ✓ | ✓ | ✓ |
| 8 hours | ✓ | ✓ | ✓ | ✓ | ✓ | ✓ | ✓ | ✓ |
| 10 hours | ✓ | ✓ | ✓ | ✓ | ✓ | ✓ | ✓ | ✓ |
| 12 hours | ✓ | ✓ | ✓ | ✓ | ✓ | ✓ | ✓ | ✓ |
| 24 hours | ✓ | ✓ | ✓ | ✓ | ✓ | ✓ | ✓ | ✓ |
| 36 hours | ✓ | ✓ | ✓ | ✓ | ✓ | ✓ | ✓ | ✓ |
| 48 hours | ✓ | ✓ | ✓ | ✓ | ✓ | ✓ | ✓ | ✓ |

| **Calf ID** | 6 | **Treat** | LID | |  |  |  |  |
| --- | --- | --- | --- | --- | --- | --- | --- | --- |
|  | **Right horn bud** | | | | **Left horn bud** | | | |
| **Time** | **cranial** | **caudal** | **medial** | **lateral** | **cranial** | **caudal** | **medial** | **lateral** |
| Baseline | 6.8 | 5.2 | 5.7 | 7.3 | 5.6 | 4.7 | 7.4 | 6.9 |
| 5 mins | NR | NR | NR | NR | NR | NR | NR | NR |
| 15 mins | NR | NR | NR | NR | NR | NR | NR | NR |
| 30 mins | NR | NR | NR | NR | NR | NR | NR | NR |
| 1.25 hour | NR | NR | NR | NR | NR | NR | NR | NR |
| 1.5 hour | NR | NR | NR | NR | ✓ | ✓ | ✓ | ✓ |
| 2 hours | ✓ | ✓ | ✓ | ✓ | 10 | 3.9 | 7 | 7 |
| 4 hours | ✓ | ✓ | ✓ | ✓ | ✓ | ✓ | ✓ | ✓ |
| 6 hours | ✓ | ✓ | ✓ | ✓ | ✓ | ✓ | ✓ | ✓ |
| 8 hours | ✓ | ✓ | ✓ | ✓ | ✓ | ✓ | ✓ | ✓ |
| 10 hours | ✓ | ✓ | ✓ | ✓ | ✓ | ✓ | ✓ | ✓ |
| 12 hours | ✓ | ✓ | ✓ | ✓ | ✓ | ✓ | ✓ | ✓ |
| 24 hours | ✓ | ✓ | ✓ | ✓ | ✓ | ✓ | ✓ | ✓ |
| 36 hours | ✓ | ✓ | ✓ | ✓ | ✓ | ✓ | ✓ | ✓ |
| 48 hours | ✓ | ✓ | ✓ | ✓ | ✓ | ✓ | ✓ | ✓ |

| **Calf ID** | 7 | **Treat** | BUP-SAIB | |  |  |  |  |
| --- | --- | --- | --- | --- | --- | --- | --- | --- |
|  | **Right horn bud** | | | | **Left horn bud** | | | |
| **Time** | **cranial** | **caudal** | **medial** | **lateral** | **cranial** | **caudal** | **medial** | **lateral** |
| Baseline | 3.7 | 6.3 | 5.6 | 7.3 | 6 | 5.7 | 3.9 | 4.6 |
| 5 mins | NR | NR | NR | NR | NR | NR | NR | NR |
| 15 mins | NR | NR | NR | NR | NR | NR | NR | NR |
| 30 mins | NR | NR | NR | NR | NR | NR | NR | NR |
| 1 hour | NR | NR | NR | NR | NR | NR | NR | NR |
| 1.5 hour | NR | NR | NR | NR | NR | NR | NR | NR |
| 2 hours | NR | NR | NR | NR | NR | NR | NR | NR |
| 4 hours | NR | NR | NR | NR | NR | NR | NR | NR |
| 6 hours | NR | NR | NR | NR | NR | NR | NR | NR |
| 8 hours | NR | NR | NR | NR | NR | NR | NR | NR |
| 10 hours | ✓ | ✓ | ✓ | ✓ | NR | NR | NR | NR |
| 12 hours | ✓ | ✓ | ✓ | ✓ | ✓ | ✓ | ✓ | ✓ |
| 24 hours | ✓ | ✓ | ✓ | ✓ | ✓ | ✓ | ✓ | ✓ |
| 36 hours | ✓ | ✓ | ✓ | ✓ | ✓ | ✓ | ✓ | ✓ |
| 48 hours | ✓ | ✓ | ✓ | ✓ | ✓ | ✓ | ✓ | ✓ |

| **Calf ID** | 8 | **Treat** | BUP-SAIB | |  |  |  |  |
| --- | --- | --- | --- | --- | --- | --- | --- | --- |
|  | **Right horn bud** | | | | **Left horn bud** | | | |
| **Time** | **cranial** | **caudal** | **medial** | **lateral** | **cranial** | **caudal** | **medial** | **lateral** |
| Baseline | 5.8 | 5.3 | 6 | 5.3 | 5 | 4.5 | 3.9 | 6.8 |
| 5 mins | NR | NR | NR | NR | NR | NR | NR | NR |
| 15 mins | NR | NR | NR | NR | NR | NR | NR | NR |
| 30 mins | NR | NR | NR | NR | NR | NR | NR | NR |
| 1 hour | NR | NR | NR | NR | NR | NR | NR | NR |
| 1.5 hour | NR | NR | NR | NR | NR | NR | NR | NR |
| 2 hours | NR | NR | NR | NR | NR | NR | NR | NR |
| 4 hours | NR | NR | NR | NR | NR | NR | NR | NR |
| 6 hours | NR | NR | NR | NR | NR | NR | NR | NR |
| 8 hours | NR | NR | NR | NR | NR | NR | NR | NR |
| 10 hours | NR | NR | NR | NR | NR | NR | NR | NR |
| 12 hours | NR | NR | NR | NR | NR | NR | NR | NR |
| 24 hours | NR | NR | NR | NR | ✓ | ✓ | ✓ | ✓ |
| 36 hours | NR | NR | NR | NR | ✓ | ✓ | ✓ | ✓ |
| 48 hours | ✓ | ✓ | ✓ | ✓ | ✓ | ✓ | ✓ | ✓ |

| **Calf ID** | 9 | **Treat** | LID |  |  |  |  |  |
| --- | --- | --- | --- | --- | --- | --- | --- | --- |
|  | **Right horn bud** | | | | **Left horn bud** | | | |
| **Time** | **cranial** | **caudal** | **medial** | **lateral** | **cranial** | **caudal** | **medial** | **lateral** |
| Baseline | 3.6 | 4.5 | 4.9 | 6.3 | 7.4 | 5.6 | 6.3 | 7.2 |
| 5 mins | NR | NR | NR | NR | NR | NR | NR | NR |
| 15 mins | NR | NR | NR | NR | NR | NR | NR | NR |
| 30 mins | NR | NR | NR | NR | NR | NR | NR | NR |
| 1 hour | ✓ | ✓ | ✓ | ✓ | ✓ | ✓ | ✓ | ✓ |
| 1.5 hour | ✓ | ✓ | ✓ | ✓ | ✓ | ✓ | ✓ | ✓ |
| 2 hours | ✓ | ✓ | ✓ | ✓ | ✓ | ✓ | ✓ | ✓ |
| 4 hours | ✓ | ✓ | ✓ | ✓ | ✓ | ✓ | ✓ | ✓ |
| 6 hours | ✓ | ✓ | ✓ | ✓ | ✓ | ✓ | ✓ | ✓ |
| 8 hours | ✓ | ✓ | ✓ | ✓ | ✓ | ✓ | ✓ | ✓ |
| 10 hours | ✓ | ✓ | ✓ | ✓ | ✓ | ✓ | ✓ | ✓ |
| 12 hours | ✓ | ✓ | ✓ | ✓ | ✓ | ✓ | ✓ | ✓ |
| 24 hours | ✓ | ✓ | ✓ | ✓ | ✓ | ✓ | ✓ | ✓ |
| 36 hours | ✓ | ✓ | ✓ | ✓ | ✓ | ✓ | ✓ | ✓ |
| 48 hours | ✓ | ✓ | ✓ | ✓ | ✓ | ✓ | ✓ | ✓ |

| **Calf ID** | 10 | **Treat** | BUP-SAIB | |  |  |  |  |
| --- | --- | --- | --- | --- | --- | --- | --- | --- |
|  | **Right horn bud** | | | | **Left horn bud** | | | |
| **Time** | **cranial** | **caudal** | **medial** | **lateral** | **cranial** | **caudal** | **medial** | **lateral** |
| Baseline | 4.5 | 5.3 | 6.1 | 4.2 | 6.2 | 5.9 | 6.1 | 7 |
| 5 mins | NR | NR | NR | NR | NR | NR | NR | NR |
| 15 mins | NR | NR | NR | NR | NR | NR | NR | NR |
| 30 mins | NR | NR | NR | NR | NR | NR | NR | NR |
| 1 hour | NR | NR | NR | NR | NR | NR | NR | NR |
| 1.5 hour | NR | NR | NR | NR | NR | NR | NR | NR |
| 2 hours | NR | NR | NR | NR | NR | NR | NR | NR |
| 4 hours | NR | NR | NR | NR | NR | NR | NR | NR |
| 6 hours | NR | NR | NR | NR | NR | NR | NR | NR |
| 8 hours | NR | NR | NR | NR | NR | NR | NR | NR |
| 10 hours | NR | NR | NR | NR | NR | NR | NR | NR |
| 12 hours | NR | NR | NR | NR | NR | NR | NR | NR |
| 24 hours | ✓ | ✓ | ✓ | ✓ | NR | NR | NR | NR |
| 36 hours | ✓ | ✓ | ✓ | ✓ | ✓ | ✓ | ✓ | ✓ |
| 48 hours | ✓ | ✓ | ✓ | ✓ | ✓ | ✓ | ✓ | ✓ |

| **Calf ID** | 11 | **Treat** | LID |  |  |  |  |  |
| --- | --- | --- | --- | --- | --- | --- | --- | --- |
|  | **Right horn bud** | | | | **Left horn bud** | | | |
| **Time** | **cranial** | **caudal** | **medial** | **lateral** | **cranial** | **caudal** | **medial** | **lateral** |
| Baseline | 11.4 | 12 | 10.9 | 6.2 | 6.2 | 11.1 | 11.9 | 9.4 |
| 5 mins | NR | NR | NR | NR | NR | NR | NR | NR |
| 15 mins | NR | NR | NR | NR | NR | NR | NR | NR |
| 30 mins | NR | NR | NR | NR | NR | NR | NR | NR |
| 1 hour | ✓ | ✓ | ✓ | ✓ | NR | NR | NR | NR |
| 1.5 hour | ✓ | ✓ | ✓ | ✓ | ✓ | ✓ | ✓ | ✓ |
| 2 hours | ✓ | ✓ | ✓ | ✓ | ✓ | ✓ | ✓ | ✓ |
| 4 hours | ✓ | ✓ | ✓ | ✓ | ✓ | ✓ | ✓ | ✓ |
| 6 hours | ✓ | ✓ | ✓ | ✓ | ✓ | ✓ | ✓ | ✓ |
| 8 hours | ✓ | ✓ | ✓ | ✓ | ✓ | ✓ | ✓ | ✓ |
| 10 hours | ✓ | ✓ | ✓ | ✓ | ✓ | ✓ | ✓ | ✓ |
| 12 hours | ✓ | ✓ | ✓ | ✓ | ✓ | ✓ | ✓ | ✓ |
| 24 hours | ✓ | ✓ | ✓ | ✓ | ✓ | ✓ | ✓ | ✓ |
| 36 hours | ✓ | ✓ | ✓ | ✓ | ✓ | ✓ | ✓ | ✓ |
| 48 hours | ✓ | ✓ | ✓ | ✓ | ✓ | ✓ | ✓ | ✓ |

| **Calf ID** | 12 | **Treat** | BUP-SAIB | |  |  |  |  |
| --- | --- | --- | --- | --- | --- | --- | --- | --- |
|  | **Right horn bud** | | | | **Left horn bud** | | | |
| **Time** | **cranial** | **caudal** | **medial** | **lateral** | **cranial** | **caudal** | **medial** | **lateral** |
| Baseline | 4.2 | 5.1 | 6 | 4.8 | 5.4 | 4.7 | 5.2 | 6.1 |
| 5 mins | ✓ | ✓ | ✓ | ✓ | ✓ | ✓ | ✓ | ✓ |
| 15 mins | ✓ | ✓ | ✓ | ✓ | ✓ | ✓ | ✓ | ✓ |
| 30 mins | ✓ | ✓ | ✓ | ✓ | ✓ | ✓ | ✓ | ✓ |
| 1 hour | ✓ | ✓ | ✓ | ✓ | ✓ | ✓ | ✓ | ✓ |
| 1.5 hour | ✓ | ✓ | ✓ | ✓ | ✓ | ✓ | ✓ | ✓ |
| 2 hours | ✓ | ✓ | ✓ | ✓ | ✓ | ✓ | ✓ | ✓ |
| 4 hours | ✓ | ✓ | ✓ | ✓ | ✓ | ✓ | ✓ | ✓ |
| 6 hours | ✓ | ✓ | ✓ | ✓ | ✓ | ✓ | ✓ | ✓ |
| 8 hours | ✓ | ✓ | ✓ | ✓ | ✓ | ✓ | ✓ | ✓ |
| 10 hours | ✓ | ✓ | ✓ | ✓ | ✓ | ✓ | ✓ | ✓ |
| 12 hours | ✓ | ✓ | ✓ | ✓ | ✓ | ✓ | ✓ | ✓ |
| 24 hours | ✓ | ✓ | ✓ | ✓ | ✓ | ✓ | ✓ | ✓ |
| 36 hours | ✓ | ✓ | ✓ | ✓ | ✓ | ✓ | ✓ | ✓ |
| 48 hours | ✓ | ✓ | ✓ | ✓ | ✓ | ✓ | ✓ | ✓ |

| **Calf ID** | 13 | **Treat** | LID |  |  |  |  |  |
| --- | --- | --- | --- | --- | --- | --- | --- | --- |
|  | **Right horn bud** | | | | **Left horn bud** | | | |
| **Time** | **cranial** | **caudal** | **medial** | **lateral** | **cranial** | **caudal** | **medial** | **lateral** |
| Baseline | 12 | 12 | 12 | 9.7 | 9.8 | 8.9 | 6.7 | 11.1 |
| 5 mins | NR | NR | NR | NR | NR | NR | NR | NR |
| 15 mins | NR | NR | NR | NR | NR | NR | NR | NR |
| 30 mins | NR | NR | NR | NR | NR | NR | NR | NR |
| 1 hour | NR | NR | NR | NR | ✓ | ✓ | ✓ | ✓ |
| 1.5 hour | ✓ | ✓ | ✓ | ✓ | ✓ | ✓ | ✓ | ✓ |
| 2 hours | ✓ | ✓ | ✓ | ✓ | ✓ | ✓ | ✓ | ✓ |
| 4 hours | ✓ | ✓ | ✓ | ✓ | ✓ | ✓ | ✓ | ✓ |
| 6 hours | ✓ | ✓ | ✓ | ✓ | ✓ | ✓ | ✓ | ✓ |
| 8 hours | ✓ | ✓ | ✓ | ✓ | ✓ | ✓ | ✓ | ✓ |
| 10 hours | ✓ | ✓ | ✓ | ✓ | ✓ | ✓ | ✓ | ✓ |
| 12 hours | ✓ | ✓ | ✓ | ✓ | ✓ | ✓ | ✓ | ✓ |
| 24 hours | ✓ | ✓ | ✓ | ✓ | ✓ | ✓ | ✓ | ✓ |
| 36 hours | ✓ | ✓ | ✓ | ✓ | ✓ | ✓ | ✓ | ✓ |
| 48 hours | ✓ | ✓ | ✓ | ✓ | ✓ | ✓ | ✓ | ✓ |

| **Calf ID** | 14 | **Treat** | BUP-SAIB | |  |  |  |  |
| --- | --- | --- | --- | --- | --- | --- | --- | --- |
|  | **Right horn bud** | | | | **Left horn bud** | | | |
| **Time** | **cranial** | **caudal** | **medial** | **lateral** | **cranial** | **caudal** | **medial** | **lateral** |
| Baseline | 11.8 | 9.7 | 8.4 | 5.4 | 5.9 | 6.3 | 8.1 | 7.9 |
| 5 mins | NR | NR | NR | NR | NR | NR | NR | NR |
| 15 mins | NR | NR | NR | NR | NR | NR | NR | NR |
| 30 mins | NR | NR | NR | NR | NR | NR | NR | NR |
| 1 hour | NR | NR | NR | NR | NR | NR | NR | NR |
| 1.5 hour | NR | NR | NR | NR | NR | NR | NR | NR |
| 2 hours | NR | NR | NR | NR | NR | NR | NR | NR |
| 4 hours | NR | NR | NR | NR | NR | NR | NR | NR |
| 6 hours | NR | NR | NR | NR | NR | NR | NR | NR |
| 8 hours | NR | NR | NR | NR | NR | NR | NR | NR |
| 10 hours | NR | NR | NR | NR | NR | NR | NR | NR |
| 12 hours | NR | NR | NR | NR | NR | NR | NR | NR |
| 24 hours | NR | NR | NR | NR | NR | NR | NR | NR |
| 36 hours | ✓ | ✓ | ✓ | ✓ | ✓ | ✓ | ✓ | ✓ |
| 48 hours | ✓ | ✓ | ✓ | ✓ | ✓ | ✓ | ✓ | ✓ |
